# A role for Arabidopsis myosins in sugar-induced hypocotyl elongation

**DOI:** 10.17912/micropub.biology.000276

**Published:** 2020-07-10

**Authors:** Damilola Olatunji, Dior R Kelley

**Affiliations:** 1 Iowa State University

**Figure 1:**
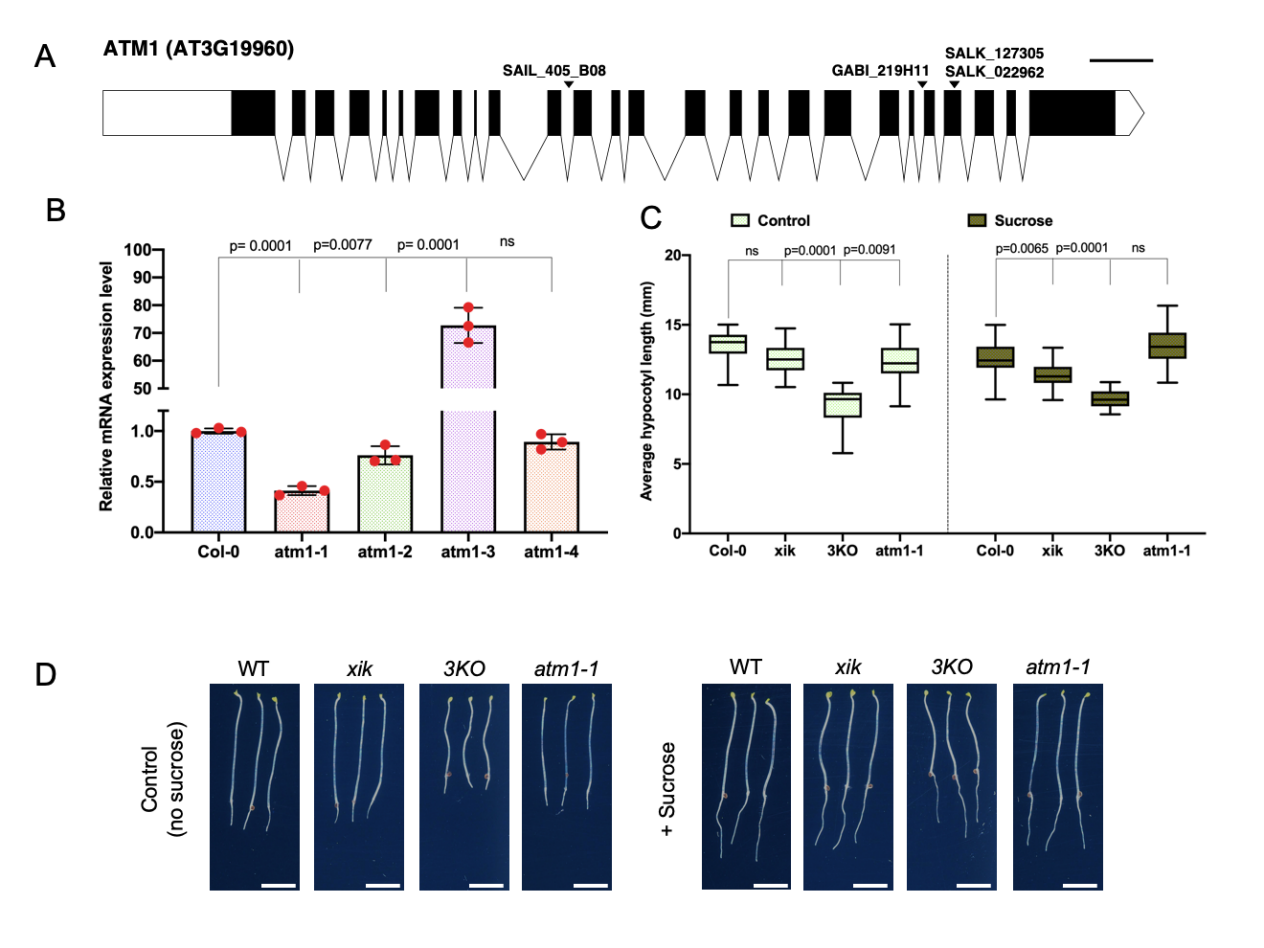
(A) Schematic gene structure of *ATM1* (AT3G19960) indicating the T-DNA insertions assayed in this study: SAIL_405_B08 *(atm1-1*), SALK_127305 *(atm1-*2), GABI_209H11 *(atm1-*3), and SALK_022962 (*atm1-4*)*.* Black boxes and lines depict exons and introns respectively. Scale bar is 100 bases. (B) *ATM1* transcript levels in different alleles (*atm1-1*, *atm1-2*, *atm1-3*, and atm*1-4*) compared to Columbia Ecotype (Col-0) assayed by quantitative PCR (qPCR). (C) Average hypocotyl length of seedlings grown on 0.5X Murashige and Skoog (MS) medium with or without 15 mM sucrose in the dark for 5 days. A total of 30-40 seedlings were evaluated and the assay was replicated three times (panel shows the results from one experiment). Error bars show standard error. P values are calculated using one- or two-way ANOVA followed by Tukey multiple comparisons; ns: not significant. (D) Hypocotyl phenotypes after 5 days of incubation in continuous darkness. Scale bar: 5mm

## Description

The ability of developing seedlings to respond and adapt to diverse environmental conditions including light is critical for their emergence and establishment (Benvenuti *et al.*, 2001; Forcella *et al.*, 2000; Salter *et al.*, 2003; Yu and Huang, 2017). Cell expansion within the hypocotyl optimizes light and energy capture by the cotyledons, and enables the transition to autotrophic status (Botterweg-Paredes *et al.*, 2020; Dowson‐Day and Millar, 1999; Oh *et al.*, 2013). Hypocotyl elongation is regulated by multiple factors including temperature, phytohormones, circadian clock and light (Dowson‐Day and Millar, 1999; Ma *et al.*, 2016; Procko *et al.*, 2014; Reed *et al.*, 2018; Yu and Huang, 2017). Endogenous and exogenous sugars are also important regulators of hypocotyl cell expansion (Lilley *et al.*, 2012; Liu *et al.*, 2011; Pfeiffer and Kutschera, 1995; Simon *et al.*, 2018b; Singh *et al.*, 2017; Zhang *et al.*, 2010, 2015). Under constant darkness, the interaction between plant hormones such as brassinosteroid and gibberellin and sugar signalling is proposed to stimulate increase in hypocotyl length (Simon *et al.*, 2018b; Zhang *et al.*, 2010, 2015). Hypocotyl phenotypes are important tools in plant biology as they have been used to screen for mutants with altered responses to light and sugar signalling and some of the identified genes have revolutionized plant research (Nakano, 2019). In multicellular organisms, actomyosin-dependent transport constitutes an essential component of the cellular structure and dynamics (Duan and Tominaga, 2018; Kurth *et al.*, 2017; Peremyslov *et al.*, 2010). The Arabidopsis genome encodes 17 myosins classified into two distantly-related groups comprising 4 class VIII myosins and 13 class XI plant myosins (Haraguchi *et al.*, 2019; Reddy and Day, 2001; Ryan and Nebenführ, 2018). The plant-specific-myosin XI has been implicated in diverse developmental processes (Cai *et al.*, 2014), but the contribution of members of class VIII myosins including myosin1/ATM1 to plant development stills remains elusive.

Here, we analyzed the role of plant myosins in sucrose-induced hypocotyl elongation under continuous darkness conditions. To start the molecular characterization of *ATM1* (AT3G19960), we analyzed four T-DNA insertional alleles (Fig. 1A), and mutations were confirmed via PCR-based genotyping. We also performed RT-qPCR to quantify *ATM1* transcripts in the four alleles, of which *atm1-1* (SAIL_405_BO8 previously described by Haraguchi *et al.*, 2014) showed reduced *ATM1* expression among these alleles compared to the wild type (Fig. 1B). Notably, *atm1-3* exhibits increased mRNA levels and may be a overexpression allele. Since *atm1-1* had the lowest *ATM1* transcript level, and was previously described as a null (Haraguchi *et al.*, 2014) we decided to use this allele for further phenotypic analysis. To understand the contribution of plant myosins in sugar-regulated hypocotyl cell expansion we performed growth assays using Col-0 (WT: wild-type), *atm1-1*, a class XI myosin single gene mutant (*xik*) and the triple knockout line (*3KO*; *xi1 xi2 xik*). This triple mutant was previously generated using SALK insertion lines in XI-K (SALK_067972; At5g20490), XI-1 (SALK_019031; At1g17580) and XI-2 (SALK_055785; At5g43900) (Prokhnevsky et. al., 2008; Valera et. al., 2010). All genotypes were grown on 0.5X Murashige and Skoog (MS) medium without or with 15 mM sucrose under constant darkness for 5 days. Under the sucrose-free condition (control), all the three myosin mutants showed reduced hypocotyl length compared to the WT plants (Fig. 1C and D), suggesting the role of myosins in cell elongation. Specifically, the *3KO* mutant was severely impaired in hypocotyl cell expansion in the absence of sucrose when compared to the single mutants, hinting that this gene family may redundantly regulate plant development. In the presence of sucrose under constant darkness, the short hypocotyl phenotype was not fully rescued when compared to the WT plants except for *atm1-1*.

Altogether our findings suggest that plant-specific myosins may be involved in sugar-regulated hypocotyl elongation under continuous darkness. These myosins have diverse subcellular localization patterns (Haraguchi *et al.*, 2014; Peremyslov *et al.*, 2012, 2008) and thus may represent a new downstream signaling output following sucrose signals within the developing shoot. Sucrose-induced hypocotyl elongation has been linked to brassinosteroid, gibberellins, phytochrome interacting factors (PIFs) and SnRK1 (Sucrose Nonfermenting Kinase 1) signaling (Laxmi *et al.*, 2004; Liu *et al.*, 2011; Simon *et al.*, 2018a; Zhang *et al.*, 2015, 2010; Zhang and He, 2015). Other proteins that are important for this process include TANG1 (Zheng *et al.*, 2015), a WD40 protein AtGHS40 (Hsiao *et al.*, 2016), a plant-specific protein SR45 (Carvalho *et al.*, 2010), and HIGH SUGAR RESPONSE 8 (HSR8) (Li *et al.*, 2007). Further studies will be needed to determine how these molecular factors work in concert or parallel to regulate skotomorphogenesis.

## Methods

**Plant materials**

Arabidopsis thaliana plants used in this study were Columbia (Col-0) ecotype: SAIL_405_B08 (*atm1-1*), SALK_127305C (*atm1-2*), GABI_219H11(*atm1-3*) and SALK_022962C (*atm1-4*) and previously described (*xik*) and *3KO* triple knockout line (*xi1 xi2 xik*) (Peremyslov *et al.*, 2012, 2010, 2008). Seeds were surfaced sterilized using 50% bleach and 0.01% Triton X-100 for 10 min and then washed five times with sterile water. Seeds were then imbibed in sterile water for 2 days at 4 °C and then transferred to 0.5X Murashige and Skoog (MS) medium plates supplemented without/with 15mM sucrose. For hypocotyl elongation, plates were incubated in the light for 6 hours prior to incubation in the dark for 5 days at 22 °C. PCR based genotyping of the mutants was performed with primers listed in Table 1 and 2X DreamTaq polymerase master mix (Thermo Fisher Scientific).

**Real time quantitative PCR (RT-qPCR)**

Plant materials were harvested from 7-day old seedlings in three biological replicates. RNA samples were extracted with ZYMO RESARCH Direct-zol RNA Miniprep Plus Kit and quality was spectrophotometrically measured with the Nanodrop. cDNA synthesis was performed with SuperScript IV Reverse Transcriptase kit according to manufacturer’s instruction. The samples were run on *BIO RAD CFX Connect* Real-Time PCR Detection System with the following components per reaction of 20µL volume: 10µL iTaq Universal SYBR Green Supermix, 0.6 µL Forward Primer F (300nM), 0.6 µL Reverse Primer F (300nM) and 3µL cDNA (5ng/ul). No cDNA samples (water) were included as negative control. Cycling conditions were 5 min at 95 °C, followed by cycles of 15 s at 95 °C, 30 s at 60 °C and 30 s at 72 °C. Data acquisition was done at the end of every cycle. The samples were prepared in three biological and two technical repeats. The Comparative CT Method (ΔΔ CT Method) was used for the analysis of Ct values whereby the amount of target, normalized to an endogenous reference and relative to a calibrator and is given by 2 –ΔΔCT.

**Hypocotyl length and statistical analysis**

Five-day old dark-grown seedlings were imaged using an Epson Perfection V600 Photo Scanner. Hypocotyl length was measured with *ImageJ*. For each experimental treatment, 30-40 seedlings were measured for each genotype. The assays were repeated three independent times with similar results. GraphPad Prism (GraphPad Software, version 8.4.2) was used for statistical analysis. To compare treatments effects on the mean value of wild type and mutants, either or one- or two-way ANOVA was performed using Tukey’s test for multiple comparison.

**Gene diagram annotation**

The diagram of *ATM1* (*AT3G19960*) was created using the Exon-Intron Graphic Maker (http://wormweb.org/exonintron) using current gene model sequence from TAIR (www.arabidopsis.org). The position of all T-DNA alleles examined was obtained from the SALK SIGnAL T-DNA express Arabidopsis Gene Mapping tool (http://signal.salk.edu/cgi-bin/tdnaexpress).

## Reagents

**Table 1**: Primers used in this study.

**Table:** 

Primers	5′ > 3′ Sequence	Purpose
SAIL_405_B08 LP	TTCGTGTGAACGTTGATTCTG	Genotyping
SAIL_405_B08 RP	TCCAGCTTGAATAGATGACGG	
SALK_127305_LP	TCCTCAAGCATCACCGTTAAC	
SALK_127305_RP	GCAGAGAGCTCAAGTGTTTGG	
GABI_219H11_LP	TAAGAGCGAGACAGAGAACCG	
GABI_219H11_RP	TCGTGGTTGGTTGGTTAGAAG	
SALK_022962_LP	GGGGAAACAGAGAGAAATTGG	
SALK_022962_RP	TTTGCTTTGGCATTAACCAAC	
Sail-LB2	GCTTCCTATTATATCTTCCCAAATTACCAATACA	
LBb1.3	ATTTTGCCGATTTCGGAAC	
GABI-8474	ATAATAACGCTGCGGACATCTACATTTT	
ATM1_qPCR_Fw	CAGACAGAGAACTGAGGAGGC	RT-qPCR
ATM1_qPCR_Rv	CATCGAACCACTGCTCTCTTCG	
AT1G13320_Fw	GCGGTTGTGGAGAACATGATACG	
AT1G13320_Rv	GAACCAAACACAATTCGTTGCTG	
